# 706. Effect of the Covid-19 Pandemic on Rates of Recurrent *Clostridiodes difficile* Infection in the Veterans Affairs System

**DOI:** 10.1093/ofid/ofab466.903

**Published:** 2021-12-04

**Authors:** Dimitri M Drekonja, Jane Zhang, Andrew R Reinink, Ruth Anway, Sean Nugent, Aasma Shaukat

**Affiliations:** 1 Minneapolis Veterans Affair Health Care System, Minneapolis, MN; 2 West Haven VA Medical Center, West Haven, Connecticut; 3 Minneapolis VA Health Care System, Minneapolis, Minnesota

## Abstract

**Background:**

*Clostridiodes difficile* infection (CDI) is common and classified as an urgent threat by the US Centers for Disease Control and Prevention. Recurrence (rCDI) occurs in 30% of cases and increases with subsequent episodes. As part of a trial of fecal microbiota transplantation vs. placebo for the prevention of rCDI, rCDI is identified using a case-finding algorithm that screens for potential cases across all Veterans Affairs facilities, a key component of which is a stool test confirming the presence of *C. difficile*. With the emergence of Covid-19 in the Unites States in early 2020, study personnel observed a decreasing number of rCDI cases. We hypothesized that Covid restrictions and fear of transmission prevented patients from coming to a VA facility to submit a confirmatory stool sample, the standard method of diagnosing rCDI. Accordingly, the algorithm was modified to also identify cases where rCDI was empirically treated, without confirmatory testing. Here we report on the prevalence of empiric treatment of rCDI during the Covid pandemic and changes in lab-conformed cases over time.

**Methods:**

Cases of potentially rCDI are identified by a weekly query of VA data, using an algorithm that includes laboratory testing results, diagnostic codes, and prescriptions. The ource database is updated daily from every VA facility, encompassing over 8 million Veterans. Potential cases are reviewed by research coordinators using the medical record to determine study eligibility. Beginning June 2020, the algorithm was adjusted to also identify patients with lab confirmation of their first CDI episode but none for their recurrence and identified those who were prescribed treatment for rCDI.

**Results:**

We observed a reduction in both the number of weekly cases (22.2 vs. 17.4; P < 0.001) which is a 22% decrease after the Covid-19 emergency declaration (figure). Post-declaration, empiric treatment was prescribed to 159 Veterans (mean, 3.3/week).

Potential cases of rCDI/week pre- and post Covid-19 pandemic declaration

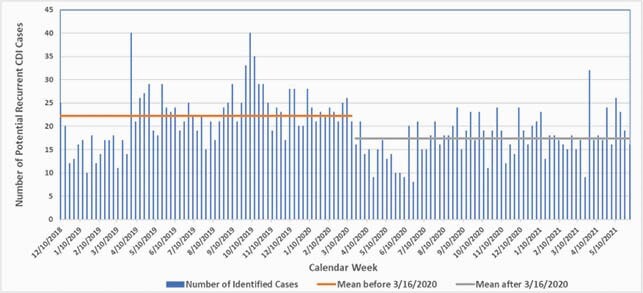

**Conclusion:**

There was a significant drop in laboratory-confirmed rCDI associated with Covid-19. Recurrent CDI was frequently empirically treated during the Covid-19 pandemic, potentially exposing many patients with non-CDI diarrhea to unnecessary antimicrobial use and its attendant risks.

**Disclosures:**

**All Authors**: No reported disclosures

